# Updating the Prevalence of *H. pylori* and Extensive Precancerous Lesions in the Stomach: Results of a Multicenter, Endoscopic Study

**DOI:** 10.3390/jcm15176841

**Published:** 2026-09-03

**Authors:** Angelo Zullo, Giulio Cozza, Guido Manfredi, Saverio Alicante, Andrea Mega, Eugenio Marconato, Salvatore Russo, Benedetta Toro, Luca De Luca, Marco Magistroni, Bastianello Germanà, Nunzia Russo, Elisabetta Martinelli, Alessandra Loiodice, Dino Vaira, Giulia Fiorini, Giuseppe Scaccianoce, Berardino D’Ascoli, Roberto Vassallo, Vincenzo De Francesco, Gianluca Esposito, Irene Ligato, Marcello Maida, Federico Bonomo, Giovanna Ruggiero, Andrea Boccuto, Francesca Galeazzi, Anna Ros, Angelo Milano, Rachele Ciccocioppo, Fabio Monica, Marco Sartori, Giulia Leonardi, Raffaele Manta

**Affiliations:** 1 Gastroenterology and Digestive Endoscopy Unit, ‘Nuovo Regina Margherita’ Hospital, 00153 Rome, Italy; 2 Department of Gastroenterology and Endoscopy, ASST Crema, 26013 Crema, Italysaverio.alicante@asst-crema.it (S.A.); 3 Gastroenterology Unit, ‘Bolzano’ Regional Hospital, 39100 Bolzano, Italy; 4 Gastroenterology and Digestive Endoscopy, ‘Azienda USL Toscana Nord Ovest’, 54100 Massa, Massa-Carrara, Italy; 5 Gastroenterology and Digestive Endoscopy, ‘ASST Santi Paolo e Carlo’, 20142 Milan, Italy; luca.deluca@unimi.it (L.D.L.);; 6 Gastroenterology and Digestive Endoscopy, ‘ULSS1 Dolomiti San Martino’ Hospital, 32100 Belluno, Italy; 7 IRCCS ‘Giovanni Paolo II’, 70124 Bari, Italy; 8 Department of Medical and Surgical Sciences, IRCCS Azienda Ospedaliero Universitaria di Bologna, University of Bologna, 40138 Bologna, Italy; 9 Interventional Gastroenterology, “Madonna Delle Grazie” Hospital, 75100 Matera, Italy; 10 Gastroenterology and Endoscopy, ‘Macchierella’ Hospital, 90138 Palermo, Italy; 11 Gastroenterology and Endoscopy Unit, ‘Riuniti’ Hospital, 71122 Foggia, Italy; 12 Department of Medical-Surgical Sciences and Translational Medicine, ‘Sant’Andrea’ Hospital, ‘Sapienza’ University, 00189 Rome, Italy; 13 Department of Medicine and Surgery, Gastroenterology Unit, ‘Umberto I’ Hospital, University of Enna ‘Kore’, 94100 Enna, Italy; 14 Digestive Endoscopy Unit, ‘Polo Ospedaliero’, Lamezia Terme, 88046 Catanzaro, Italy; 15 Department of Surgical, Gastroenterological, and Oncological Sciences, University of Padua, 35122 Padua, Italy; 16 Gastroenterology and Endoscopy Unit, ‘SS. Annunziata’ Hospital, University of Chieti, 66100 Chieti, Italy; 17 Gastroenterology and Digestive Endoscopy, ‘Cattinara’ Academic Hospital, 34127 Trieste, Italy; 18 Gastroenterology and Digestive Endoscopy, ‘Azienda USL Toscana Nord Ovest’, 57100 Livorno, Italy

**Keywords:** atrophy, gastric precancerous lesion, *Helicobacter pylori*, intestinal metaplasia, prevention, stomach

## Abstract

**Background/Objectives**: *H. pylori* is the main preventable risk factor for gastric cancer through the development of extensive precancerous lesions, namely mucosal atrophy and intestinal metaplasia. **Methods**: This multicenter, nationwide study enrolled consecutive patients who underwent their first upper gastrointestinal endoscopy with standard biopsies. Clinical, endoscopic and histological data were gathered for statistical analysis using both univariate and multivariate analyses. **Results**: Data from 775 patients were collected, and *H. pylori* infection was detected in 172 (22.2%) cases. The infection prevalence significantly increased with age, and it was significantly higher in patients aged ≥50 years compared to younger patients (26.2% vs. 13.7%; *p* < 0.001; OR: 2.23; 95% CI = 1.48–3.36). The prevalence of extensive gastric precancerous lesions was 2.6%. The multivariate analysis showed that *H. pylori* infection (OR: 4.77; 95% CI = 1.51–15; *p* = 0.007), family history of gastric cancer (OR: 4.86; 95% CI = 1.36–15; *p* = 0.007), and age (OR: 1.07; 95% CI = 1.02–1.13; *p* = 0.007) independently increased the risk of these lesions. **Conclusions**: Data provided by this large study on the endoscopic prevalence of both *H. pylori* infection and extensive precancerous lesions in the stomach may be useful for designing preventive strategies aimed at reducing gastric cancer mortality.

## 1. Introduction

Gastric cancer prevalence was estimated to remain stable—if not increased—until 2035 in different Western countries [1]. Classified as a type I carcinogen by the IARC [2], *H. pylori* is the main preventable risk factor triggering and maintaining the carcinogenetic cascade in the stomach [3]. It has been calculated that 75% of gastric cancer cases are attributable to this infection [4]. Indeed, *H. pylori*-induced chronic active inflammation on gastric mucosa leads to the development of precancerous lesions, namely mucosal atrophy and intestinal metaplasia [5], which represent the ‘point of no return’ [6]. In more detail, when these lesions extensively affect the gastric mucosa, which is staged as classes III-IV according to the OLGA (Operative Link for Gastritis Assessment) or OLGIM (Operative Link for Gastric Intestinal Metaplasia assessment) classifications, the risk of carcinoma development is distinctly increased [7]. Of note, a large, long-term follow-up study on more than 7 thousand patients found that gastric cancer develops in 19.1 and 41.2 per 1000 patients/year among those with OLGA III and OLGA IV gastric atrophy, respectively, and only in 0.03–1.48 patients/year when lower OLGA stages are present [8]. Moreover, a recent meta-analysis of eight studies showed that OLGA III–IV stages (RR: 32.31, 95% CI 9.14–114.21) and OLGIM III–IV stages (RR: 12.38, 95% CI 5.75–26.65) were associated with increased relative risk of developing gastric cancer in comparison to their 0–II categories [7]. Therefore, patients diagnosed with stage III-IV OLGA-OLGIM deserve scheduled endoscopic follow-up, according to the current guidelines [9,10].

Fortunately, the prevalence of *H. pylori* infection is decreasing in several developed countries due to both widespread implementation of eradication therapies in clinical practice and a progressive improvement in socio-economic status [11]. Consequently, assessing the prevalence of such an infection in routine practice provides clinically relevant information. Similarly, updating the prevalence of extensive precancerous lesions in the stomach, and also exploring their potential predictive factors, allows assessment of the burden of patients who require appropriate follow-up to prevent or reduce mortality from gastric cancer. Beyond *H. pylori* infection, other risk factors for cancer development in the stomach were identified. Family history of gastric cancer, smoking habit, and male sex were reported to be independent risk factors for the development of both precancerous lesions on gastric mucosa and carcinoma. Indeed, first-degree relatives of gastric cancer patients have a higher risk of developing such neoplasia, with adjusted odds ratios ranging from 1.8 (95% CI = 1.6–2.0) to 9.9 (95% CI = 6.5–15), as calculated in a meta-analysis [12]. Similarly, a meta-analysis of 40 studies found that smoking was associated with approximately 1.44-fold (95% CI = 1.17–1.78) higher risk of gastric cancer [13], and by 2.05-fold (95% CI = 1.47–2.85) higher risk of developing precancerous lesions on gastric mucosa [14]. Moreover, the incidence of gastric cancer has been consistently reported to be higher in males than in females in all geographic areas, including those at high (≥10 cases per 100,000 persons/year), intermediate, and low (≤5 cases per 100,000 persons/year) incidence of neoplasia [15]. The protective role of sex hormones and hormone replacement in post-menopausal females has been well-documented [16]. On the contrary, the role of alcohol intake appears to be controversial, and only heavy alcohol consumption seems to be an independent risk factor [17].

To obtain reliable data on the prevalence of both *H. pylori* infection and extensive precancerous lesions in the gastric mucosa, we designed this cross-sectional study among patients who underwent their first upper gastrointestinal endoscopy (UGIE) in routine practice.

## 2. Materials and Methods

In this multicenter, nationwide study, all consecutive adult patients who underwent UGIE were invited to participate in this survey and consent to the use of their clinical information for scientific purposes in an anonymous manner. For this purpose, signed informed consent was obtained for all endoscopic procedures. Inclusion criteria were (a) first endoscopic examination, irrespective of indication and (b) subjects born and living in Italy. Exclusion criteria included (1) previous UGIE for any reason; (2) previous therapy for *H. pylori* infection; (3) therapy with a proton pump inhibitor and/or antibiotics in the last 14 days; (4) contraindication to take standard gastric biopsy sampling; (5) liver cirrhosis; (6) kidney failure; and (7) UGIE performed in an urgent setting.

All consenting patients underwent UGIE, and 5 standard gastric biopsies were taken, with 2 antral and 1 *incisura angularis* specimen put in one vial, and 2 gastric body biopsies in another vial, according to the updated Sydney System [18]. The specimens were fixed in formaldehyde and transported under standard conditions and histologically examined in the respective Pathology Unit to search for *H. pylori* infection and gastritis staging. *H. pylori* infection was considered present when bacteria were detected at histological assessment on hematoxylin–eosin or modified Giemsa staining in doubtful cases. Extensive precancerous lesions on gastric mucosa were consistently diagnosed when atrophy and/or intestinal metaplasia involved both antral and gastric body mucosa [5,10].

Both endoscopic examinations and pathology assessment were performed as part of routine practice. The participating centers were required to enroll 50 consecutive patients, but no fewer than 25 were to be included in the study. In each center, patients’ clinical, endoscopic, and histological data were anonymously collected in a shared Excel database created ad hoc for this study and then gathered in a single center for statistical analysis. The collected clinical features included the main symptom prompting UGIE, such as dyspepsia, gastro-esophageal reflux symptoms, and alarm symptoms (anemia, gastrointestinal bleeding, dysphagia, persistent vomiting, and weight loss). The self-reported information on the presence of a first-degree relative with gastric cancer in the family and active smoking habit was collected during a routine personal interview before UGIE.

### 2.1. Institutional Review Board Statement

Since experimental drugs were not administered and no extra examinations were performed, no additional costs or procedures for patients were required. No patient identification was allowed, no funds were received, and formal approval by the Institutional Review Board was waived. The study was performed according to good clinical practices and the Declaration of Helsinki.

### 2.2. Statistical Analysis

Frequencies, percentages, mean values with standard deviations, and 95% confidence intervals (CIs) were calculated for all observations. Univariate analyses were performed using the Chi-squared test and T-test, as appropriate. A multiple logistic regression was used to assess the relationship between extensive precancerous lesions on gastric mucosa and age, gender, current smoking habit, main symptom, first-degree history of gastric cancer, and *H. pylori* infection. Odds ratios (ORs) and 95% confidence intervals (CIs) were calculated. A *p* value less than 0.05 was considered statistically significant.

## 3. Results

### 3.1. Clinical Data

**Figure 1:**
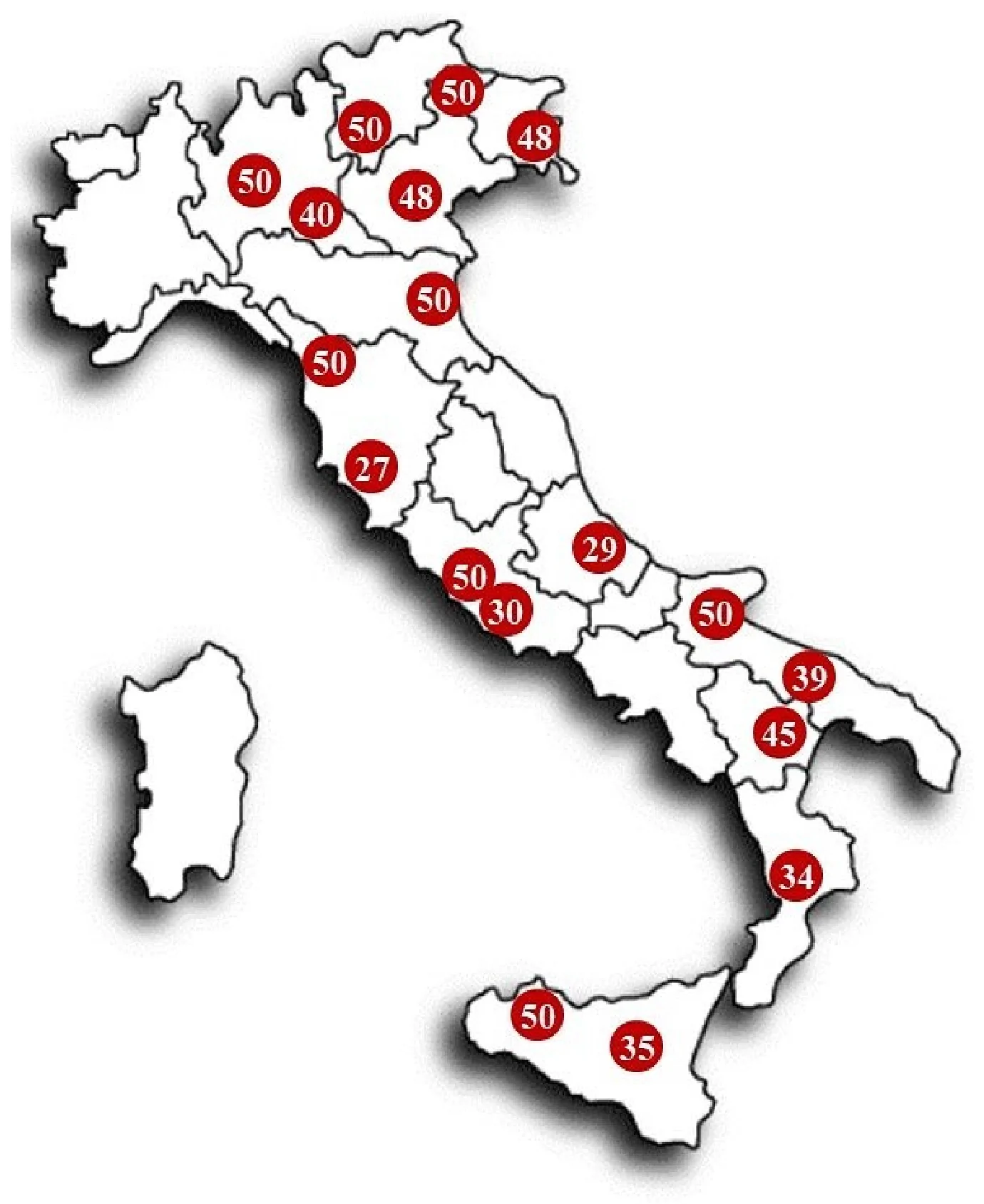
Distribution of participating centers and the number of enrolled patients.

A total of 18 centers, including 11 community hospitals and 7 academic hospitals, distributed throughout the country participated in the study (Figure 1). Overall, data from 775 patients were eventually collected. The mean age was 55.3 ± 15.6 years (range: 18–90); there were 324 (41.8%) males, 186 (24%) smokers, and 71 (9%) with a first-degree history of gastric cancer (N = 68) or esophageal cancer (N = 3). The main indications for UGIE were dyspepsia (N = 323; 41.7%), gastro-esophageal reflux symptoms (N = 270; 34.8%), and alarm symptoms (N = 169; 21.8%), whilst the 13 remaining patients underwent endoscopy for other indications.

### 3.2. H. pylori Prevalence

Overall, *H. pylori* infection was detected in 172 (22.2%) patients, including 165 with bacteria in the antrum (with or without in the corpus) and 7 with bacteria confined only to the gastric body mucosa. The prevalence of infection according to the patients’ age is provided in Table 1. In univariate analysis, the prevalence of infection in patients aged ≥50 years (138 out of 527) was double that of younger patients (34 out of 248), with the difference being statistically significant (26.2% vs. 13.7%; *p* < 0.001). The infection prevalence in patients with alarm symptoms (47 out of 169) tended to be higher than in those with reflux symptoms (54 out of 270) (27.1% vs. 20%; *p* = 0.059), but did not significantly differ from dyspeptic patients (68 out of 323; 21.1%; *p* = 0.1). No differences emerged between male (80 out of 324) and female (92 out of 451) patients (24.7% vs. 20.4%; *p* = 0.15), nor between first-degree relatives of gastric cancer patients (16 out of 68) and patients without (156 out of 707) a family history of gastric cancer (23.5% vs. 22.1%; *p* = 0.4)

The multivariate analysis confirmed that the infection prevalence significantly increases only with age (OR: 1.02; 95% CI = 1.01–1.04; *p* = 0.006) and that the risk was distinctly higher in patients aged ≥50 years (OR: 2.23; 95% CI = 1.48–3.36; *p* = 0.001).

### 3.3. Gastric Precancerous Lesions

Overall, the extension of precancerous lesions was properly staged according to the OLGA/OLGIM systems and clearly reported in the pathology reports of 544 patients from 15 centers (11 routinely provided reports, and 4 inconsistently provided reports), whilst the information was lacking in the remaining three centers. Overall, there were 14 (2.6%) out of 544 patients in whom the extension of atrophy/metaplasia on gastric mucosa was staged as grade III, and none as grade IV. In univariate analysis, the prevalence of extensive precancerous lesions was associated with age, first-degree family history of gastric cancer, and *H. pylori* infection, but not with current smoking habit (Table 2). In detail, the presence of extensive precancerous lesions on the gastric mucosa was detected exclusively in patients aged ≥50 years (range: 55–79). The multivariate analysis showed that *H. pylori* infection (OR: 4.77; 95% CI = 1.51–15; *p* = 0.005), family history of gastric cancer (N = 50) (OR: 4.86; 95% CI = 1.36–15; *p* = 0.04), and age (OR: 1.07; 95% CI = 1.02–1.13; *p* < 0.001) independently increased the risk of extensive precancerous lesions in the stomach, whilst both smoking habit and gender did not.

## 4. Discussion

Despite its decreasing incidence, gastric cancer remains the fifth leading cause of cancer-related death worldwide, representing a major global public health problem [19]. Indeed, it was estimated that the number of new gastric cancer cases in Europe will increase from 136,000 in 2022 to 174,000 in 2050, and gastric cancer-related deaths from 95,400 to 128,000 [20]. Furthermore, the 5-year survival rate from gastric cancer remains poor and is estimated to be 25% across Europe [21].

There are some European countries where the age-standardized incidence rate in men is as high as 19 in Latvia, 18.4 in Lithuania, 18 in Portugal and 17.5 in Estonia per 100,000 population [22]. Moreover, some geographic areas with an increased incidence of gastric cancer may be present even in a country at low-intermediate risk. For instance, in Northern Italy, there are some regions where gastric cancer incidence is as high as 18.5–34.8 per 100,000 population [23,24].

It has been widely reported that gastric cancer develops more frequently in males than in females, with a male-to-female ratio of age-standardized incidence rates of 2.20 [25]. Such a phenomenon was attributed to a protective role of estrogens. Indeed, the incidence of such neoplasia is lower (OR: 0.74; 95% CI = 0.63–0.86) during fertility and tends to increase in post-menopausal females, in whom estrogen supplementation reduces the risk (OR: 0.77; 95% CI = 0.64–0.92) [16]. The role of estrogen in gastric neoplasia is quite complex and depends on the type of estrogen receptor expressed on the neoplastic cells. The expression of the ERα66 receptor correlates with reduced survival, ERβ3 with increased survival, ERα36 with increased metastasis risk, and ERβ2 with reduced tumoral invasiveness [26].

Based on all these observations, different measures aiming to reduce gastric cancer incidence and/or mortality are needed [27]. Of note, the implementation of a population-based *H. pylori* screen-and-treat program for gastric cancer prevention was proposed in a recent IARC Working Group Report, especially in countries or geographic areas at high-intermediate risk for gastric cancer [15]. On the other hand, the identification and scheduled follow-up of patients with extensive gastric precancerous lesions would allow diagnosis of early neoplastic lesions—potentially amenable to endoscopic removal [28]—and reduce mortality for a cancer with a dismal prognosis.

In such a context, data collected in the present nationwide multicenter study that recruited 775 consecutive patients who underwent their first UGIE provide some clinically relevant information. Specifically, we computed that the prevalence of this infection in patients with upper gastrointestinal symptoms is 22%. Of note, our data showed that only 1 in 10 patients aged less than 50 years was infected, whereas more than two or three cases were observed in patients older than 50 or 60 years, respectively. This finding would confirm the cohort effect for the prevalence of *H. pylori* in our country, as observed in other Western geographic areas [29]. On the other hand, our data showed that *H. pylori* prevalence did not differ between patients with dyspeptic or reflux symptoms, suggesting that infection should be adequately sought during endoscopy, irrespective of the main complained symptom. Data from an Italian nationwide study performed in 2012 found higher *H. pylori* prevalence, with a value of 33.9% out of 1054 patients who underwent UGIE [30].

Precancerous lesions on gastric mucosa are consistently recognized as at risk of cancer development [5]. Taking into account data from 166 studies with 1,759,716 patients who underwent upper endoscopy worldwide, a recent systematic review estimated that the prevalence of atrophy on gastric mucosa (at least in one gastric site) was 18.0% (95% CI = 14.1–22.8%) and that of intestinal metaplasia was 15.6% (95% CI = NA), with values significantly higher in regions of high gastric cancer incidence compared to those of medium and low incidence [31]. Besides their presence, the extension of these precancerous lesions in the stomach plays a pivotal role in gastric cancer development. In detail, when atrophy and/or intestinal metaplasia involve both antral and gastric body mucosa—namely extensive precancerous lesions (OLGA-OLGIM stages III or IV)—the risk of gastric cancer onset is distinctly high [10].

Regarding these lesions, our data showed that the prevalence of extensive gastric mucosa involvement, a feature that warrants scheduled follow-up according to the European guidelines [10], was less than 3%. Of note, the presence of *H. pylori* infection, being a first-degree relative of cancer patients, and older age are independent factors associated with this condition, with no cases found in patients less than 50 years old in our series. In a previous nationwide Italian endoscopic study conducted two decades ago, the prevalence of extensive precancerous lesions was found in 3% among 679 patients [32]. Therefore, in our country, the prevalence of such a condition remained substantially stable. When coupling data on the prevalence of *H. pylori* infection with that of extensive precancerous lesions on gastric mucosa, as we computed in this study, the proposed strategy for test-and-treat *H. pylori* infection to prevent gastric cancer probably could be more cost-effective in subjects aged ≥50 years in our country. Of note, data from a Polish study showed that the prevalence of extensive atrophy and metaplasia on gastric mucosa peaked at ages 51–53 [33]. Similarly, in a recent study conducted in a single Italian center, the presence of extensive precancerous lesions was detected only in patients aged over 55 years with *H. pylori* infection [34]. Moreover, it was found that age ≥60 years is associated with a 2.28-fold increased risk of extensive precancerous lesions on gastric mucosa compared to younger subjects [35]. Therefore, it could be worthwhile to assess in future studies the cost-effectiveness of either a test-and-treat strategy for *H. pylori*-positive patients or UGIE screening in dyspeptic patients older than 55 years in areas at intermediate risk of gastric cancer. Family history of gastric cancer is a known risk factor for the development of cancer in the stomach [12]. Data from a recent systematic review of 21 studies found that the risk of gastric cancer onset was 2.92 (95% CI = 2.4–3.5) times higher in the first-degree relatives than in controls [36]. Moreover, a recent study showed that the first-degree relatives of gastric cancer patients are at increased risk for *H. pylori* infection and gastric precancerous conditions on gastric mucosa, regardless of symptoms or UGIE indications [37].

This study has several strengths and potential limitations. Certainly, the recruitment of only naïve cases across several centers and the standard biopsy sampling with histologic examination of gastric mucosa make our data very robust. Therefore, the prevalence of histological detection of both *H. pylori* infection and gastric precancerous lesions that we computed in this nationwide survey was reliable. A limitation is that the findings of the present study constitute only an endoscopic estimate of *H. pylori* and advanced precancerous lesions and not necessarily the true prevalence in the general population. Since the sensitivity of histology in effectively detecting *H. pylori* may be reduced in the presence of extensive intestinal metaplasia on gastric mucosa, the prevalence of *H. pylori* infection (57%) we computed in the 14 patients with extensive precancerous lesions could be underestimated. Finally, the small sample size of patients with extensive precancerous lesions on the gastric mucosa could have affected the accuracy of the odds ratio for the predictive factors.

## 5. Conclusions

In conclusion, the data provided by this large nationwide study on the prevalence of both *H. pylori* infection and extensive precancerous lesions in the stomach are consistent and could be useful for designing preventive strategies aimed at reducing gastric cancer mortality. Despite a similar prevalence of *H. pylori* infection between males and females, the lower incidence of gastric cancer in females could be due to the protective role of estrogens. Further studies are needed to confirm our findings.

## Figures and Tables

**Table 1:** Prevalence of *H. pylori* according to different age groups.

Age (yrs)	N of Patients	Positive (%)
≤30	71	8 (11.2)
31–39	70	9 (12.8)
40–49	107	17 (15.8)
50–59	173	39 (22.5)
60–69	204	61 (29.9)
>70	150	38 (25.3)

**Table 2:** Variables associated with extensive precancerous lesions on gastric mucosa.

Variable	Present (N = 14)	Absent (N = 530)	*p* Value
Male/Female	5/9	229/301	0.6
Mean age ± SD; yrs	68.6 ± 7.8	55.9 ± 15.7	<0.001
Family history of gastric cancer; y/n	4/10	46/484	0.04
Smoking habit; y/n	2/12	126/404	0.4
*H. pylori*; y/n	8/6	117/413	0.005

## Data Availability

Data are available upon reasonable request.

## Abbreviations

The following abbreviations are used in this manuscript:

OLGA	Operative Link for Gastritis Assessment
OLGIM	Operative Link for Gastric Intestinal Metaplasia Assessment
UGIE	Upper Gastrointestinal Endoscopy
IARC	International Agency for Research on Cancer
